# Adverse events of special interest following the use of BNT162b2 in adolescents: a population-based retrospective cohort study

**DOI:** 10.1080/22221751.2022.2050952

**Published:** 2022-03-21

**Authors:** Francisco Tsz Tsun Lai, Gilbert T. Chua, Edward Wai Wa Chan, Lei Huang, Mike Yat Wah Kwan, Tiantian Ma, Xiwen Qin, Celine Sze Ling Chui, Xue Li, Eric Yuk Fai Wan, Carlos King Ho Wong, Esther Wai Yin Chan, Ian Chi Kei Wong, Patrick Ip

**Affiliations:** a Centre for Safe Medication Practice and Research, Department of Pharmacology and Pharmacy, Li Ka Shing Faculty of Medicine, The University of Hong Kong, Hong Kong Special Administrative Region, People’s Republic of China; b Laboratory of Data Discovery for Health (D^2^4H), Hong Kong Science Park, Hong Kong Science and Technology Park, Hong Kong Special Administrative Region, People’s Republic of China; c Department of Paediatrics and Adolescent Medicine, School of Clinical Medicine, Li Ka Shing Faculty of Medicine, The University of Hong Kong, Hong Kong Special Administrative Region, People’s Republic of China; d Department of Paediatrics and Adolescent Medicine, Hong Kong Children’s Hospital, Hong Kong Special Administrative Region, People’s Republic of China; e Department of Paediatrics and Adolescent Medicine, Princess Margaret Hospital, Hong Kong Special Administrative Region, People’s Republic of China; f School of Nursing, Li Ka Shing Faculty of Medicine, The University of Hong Kong, Hong Kong Special Administrative Region, People’s Republic of China; g School of Public Health, Li Ka Shing Faculty of Medicine, The University of Hong Kong, Hong Kong Special Administrative Region, People’s Republic of China; h Department of Medicine, School of Clinical Medicine, Li Ka Shing Faculty of Medicine, The University of Hong Kong, Hong Kong Special Administrative Region, People’s Republic of China; i Department of Family Medicine and Primary Care, School of Clinical Medicine, Li Ka Shing Faculty of Medicine, The University of Hong Kong, Hong Kong Special Administrative Region, People’s Republic of China; j Research Department of Practice and Policy, School of Pharmacy, University College London, London, UK

**Keywords:** Chinese, coronavirus, immunization, reactogenicity, SARS-CoV-2

## Abstract

Accruing evidence suggests an increased risk of myocarditis in adolescents from messenger RNA COVID-19 vaccines. However, other potential adverse events remain under-researched. We conducted a retrospective cohort study of adolescents aged 12–18 with a territory-wide electronic healthcare database of the Hong Kong population linked with population-based vaccination records and supplemented with age- and sex-specific population numbers. Two age- and sex-matched retrospective cohorts were formed to observe 28 days following the first and second doses of BNT162b2 and estimate the age- and sex-adjusted incidence rate ratios between the vaccinated and unvaccinated. Thirty AESIs adapted from the World Health Organization’s Global Advisory Committee on Vaccine Safety were examined. Eventually, the first-dose cohort comprised 274,881 adolescents (50.25% received the first dose) and the second-dose cohort 237,964 (50.29% received the second dose). Ninety-four (34.2 per 100,000 persons) adolescents in the first-dose cohort and 130 (54.6 per 100,000 persons) in the second-dose cohort experienced ≥1 AESIs. There were no statistically significant differences in the risk of any AESI associated with BNT162b2 except myocarditis [first-dose cohort: incidence rate ratio (IRR) = 9.15, 95% confidence interval (CI) 1.14–73.16; second-dose cohort: IRR = 29.61, 95% CI 4.04–217.07] and sleeping disturbances/disorders after the second dose (IRR = 2.06, 95% CI 1.01–4.24). Sensitivity analysis showed that, with myocarditis excluded as AESIs, no significantly elevated risk of AESIs as a composite outcome associated with vaccination was observed (*P *= 0.195). To conclude, the overall absolute risk of AESIs was low with no evidence of an increased risk of AESIs except myocarditis and sleeping disturbances/disorders.

The coronavirus disease 2019 (COVID-19) pandemic incurs a huge burden to individuals of all age ranges in maintaining good health [1]. Albeit rarer in younger individuals compared with adults, a severe acute respiratory syndrome coronavirus-2 (SARS-CoV-2) infection could possibly lead to serious complications such as multiple organ failure and mortality [2]. Accordingly, the emergency use of a variety of vaccines has been extended to adolescents worldwide [3]. Of note, BNT162b2, one of the most widely used messenger RNA (mRNA) COVID-19 vaccines, is currently used to vaccinate adolescents aged as young as 12 years in numerous countries [4]. In fact, a further extension to younger age groups has been approved to include children as young as five [5].

Nevertheless, the increased risk of myocarditis has been generated with regard to the use of mRNA vaccines and BNT162b2 in particular [6–8]. Case reports and studies comparing the incidence of vaccinated individuals with the background incidence all suggest an elevated risk [9]. A large cohort study of nearly one million individuals [8] has estimated a threefold-increased risk of myocarditis among vaccinated individuals compared with the unvaccinated despite a very low absolute risk, which is consistent with findings from other studies as well [10,11]. There was also a descriptive study on the increased number of myocarditis reports in adolescents using the nationwide passive surveillance system monitoring a range of adverse events [12]. A more recent Danish cohort study, however, did not identify such a risk associated with BNT162b2 but with mRNA-1273 [13]. This widely observed elevated risk has received widespread public attention and aroused concerns over the safety of mRNA vaccines in adolescents overall. Certain adverse events with rare incidence may be difficult to capture in randomized controlled trials on a typically limited number of participants. Nevertheless, post-marketing pharmacovigilance data on the use of BNT162b2 in adolescents remain scant.

Since 14 June 2021, the age limit for BNT162b2 vaccination in Hong Kong has been lowered to 12 years or older [14]. As of 30 September 2021, more than 200,000 adolescents aged under 18 had received the first dose of BNT162b2 [14]. The SARS-CoV-2 infection rate in Hong Kong has remained low, with the daily number of confirmed COVID-19 cases maintained below 200 cases and the total cumulative number of cases kept under 12,500 out of a population of over seven million [15]. Since 16 August 2021 (up to 30 September 2021), there were zero local cases recorded in the daily reports [15]. This low infection rate is facilitative of the pharmacovigilance of BNT162b2 because adverse effects following vaccination would be unlikely induced by a COVID-19 infection. This population-based cohort study aims to describe and compare a range of adverse events of special interest in adolescents between the vaccinated and unvaccinated using a territory-wide electronic health record database with linkage to local vaccination records.

## Materials and methods

### Study design

A retrospective cohort design was adopted to examine the incidence rate ratio (IRR) of vaccination-related adverse events of special interest (AESI) within a 28-day observation period between the vaccinated and the unvaccinated.

### Data source

Territory-wide vaccination records of adolescents aged 12–18 were provided by the Department of Health (DH) of the Hong Kong Government. They were then linked with electronic health records provided by the Hospital Authority (HA), the sole provider of public inpatient services and specialist ambulatory clinics as well as a major provider of public general outpatient services, by matching the encrypted Hong Kong Identity Card number between the two databases. Our database contained essential information for the pharmacovigilance of COVID-19 vaccines such as clinical diagnoses, medication use, healthcare utilization, etc. AESIs were identified using this comprehensive database which has been used to conduct population-based COVID-19 vaccines pharmacovigilance studies on various outcomes [16–24, http://dx.doi.org/10.1111/joim.13453]. We further included adolescents without any vaccination record or healthcare utilization record in Hong Kong (unobserved in the above two data sources) from the age- and sex-specific population numbers provided by the Census and Statistics Department (C&SD) as of mid-2021 [25].

This study was approved by the Institutional Review Board of the University of Hong Kong/Hospital Authority Hong Kong West (UW 21–149 and UW 21-138) and the Department of Health Ethics Committee (LM 21/2021).

### Cohort selection and formation

Two retrospective cohorts were formed, with the observation periods starting from the first dose and from the second dose of BNT162b2, respectively. Both of these cohorts adopted the unvaccinated individuals as the comparison group to estimate the IRR. We retrieved the vaccination records of individuals aged 12–18 during the study period from DH and merged them with data of patients who received HA services from 1 January 2018 to 30 September 2021. CoronaVac recipients, consisting of those aged 18 years who chose to receive CoronaVac or clinical trial participants, were excluded from the merged cohort. For the second-dose cohort, those who had received only one dose were excluded. According to the age- and sex-specific population sizes provided by the C&SD, we further included individuals unobserved in our databases who were unvaccinated and did not use any public healthcare services by the HA since 2018. These individuals were operationalized as additional rows in the datasets coded as unvaccinated without any AESI.

Age (in years) and sex were then used to randomly match the unvaccinated with the vaccinated at the ratio of one to one by randomly selecting an adolescent of the same age and sex from the vaccinated adolescent(s) for each unvaccinated adolescent without replacement, with the vaccinated considerably outnumbering the unvaccinated. The vaccination dates (first or second dose for the respective cohort) were then mapped to the matched unvaccinated individuals as the index date, based on which the individual follow-up periods were determined. Accordingly, we removed those who died before the index date, were hospitalized on the index date (in the unvaccinated group) or had any AESI records before the index date. This approach has been used in previous large-scale pharmacovigilance studies as well [17,20,21].

### Outcome: adverse events of special interest

The selection of adverse events was based on the World Health Organization’s Global Advisory Committee on Vaccine Safety (GACVS), [26] from which a list of 30 AESIs were adopted to identify those who were diagnosed with any of the listed potential adverse reactions using the International Classification of Diseases, Ninth Revision (ICD-9) and International Classification of Primary Care (ICPC), except mortality and COVID-19, to define the primary composite outcome of this study (please see eTable 1 for the codes for the operationalization of AESI), i.e. any AESI. The observation period started from the index date and ended with 28 days after receiving the vaccine, mortality, receiving the second dose (only applicable to the first-dose cohort), or 30 September 2021 (end of data availability), whichever came earliest. Analysis on eight sub-categories of the AESI, namely, autoimmune diseases, cardiovascular system diseases, circulatory system diseases, hepato-renal system diseases, nerves and central nervous system diseases, skin and mucous membrane, bone and joints system diseases, respiratory system diseases, and diseases of other systems, as well as each specific AESI were conducted as the secondary outcomes.

### Exposure: vaccinated versus unvaccinated

Receiving BNT162b2 (one dose or two doses for the two respective cohorts) was compared with being unvaccinated as the exposure of this study given that BNT162b2 was the only approved vaccine for distribution for residents aged 12 or above in Hong Kong (as of the last day of available data) [27].

### Statistical analysis

Poisson regression models were performed to generate age- and sex-adjusted IRR to examine the association between vaccination and AESI. The same analyses were replicated on all sub-categories of AESI and each specific AESI as secondary outcomes. As a sensitivity analysis, we repeated the analysis on the primary outcome (an AESI) in the second-dose cohort with myocarditis removed from the list of AESIs in the operationalization to infer about the importance of myocarditis in the potential elevation of AESI risks.

All analyses were conducted using the R (version 4.1.1) statistical environment.

## Results

As of 30 September 2021, a total of 252,399 (63.61%) of the 396,800-adolescent population were vaccinated at least one dose, with 190,400 (56.87%) having received the second dose.

**Figure 1.**
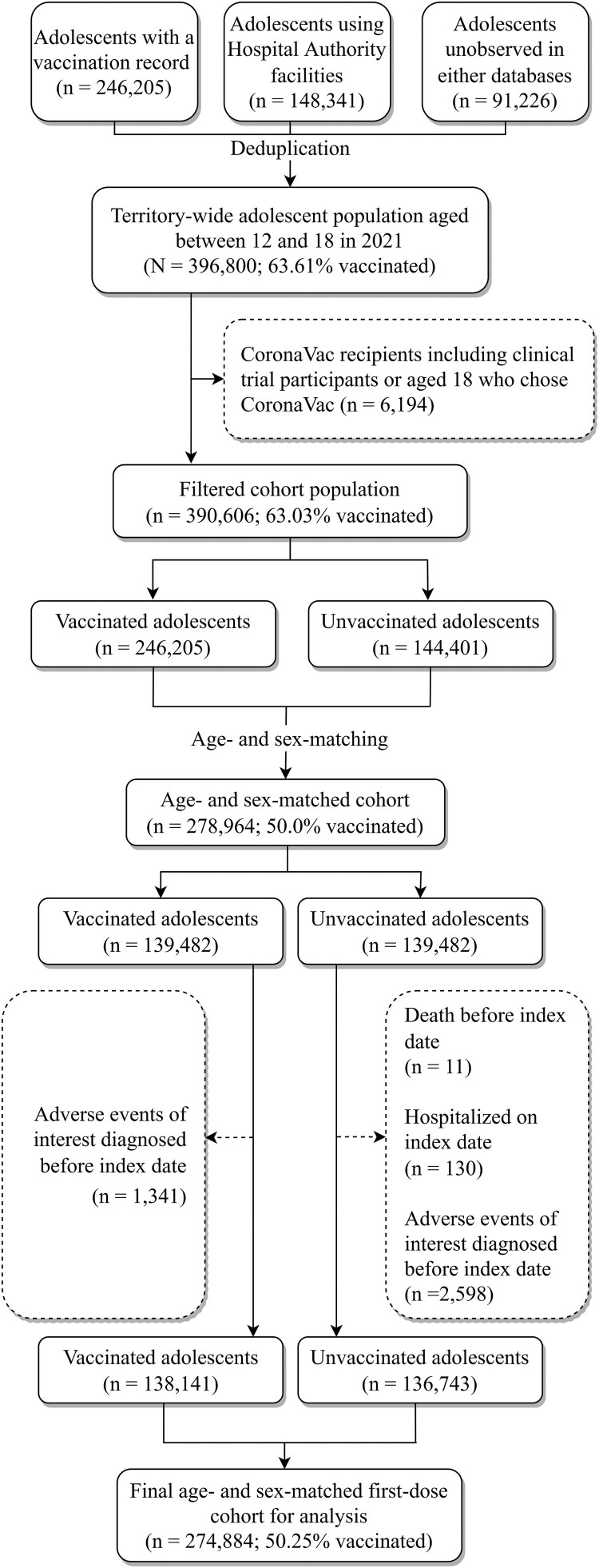
Flowchart of first-dose cohort selection.

**Figure 2.**
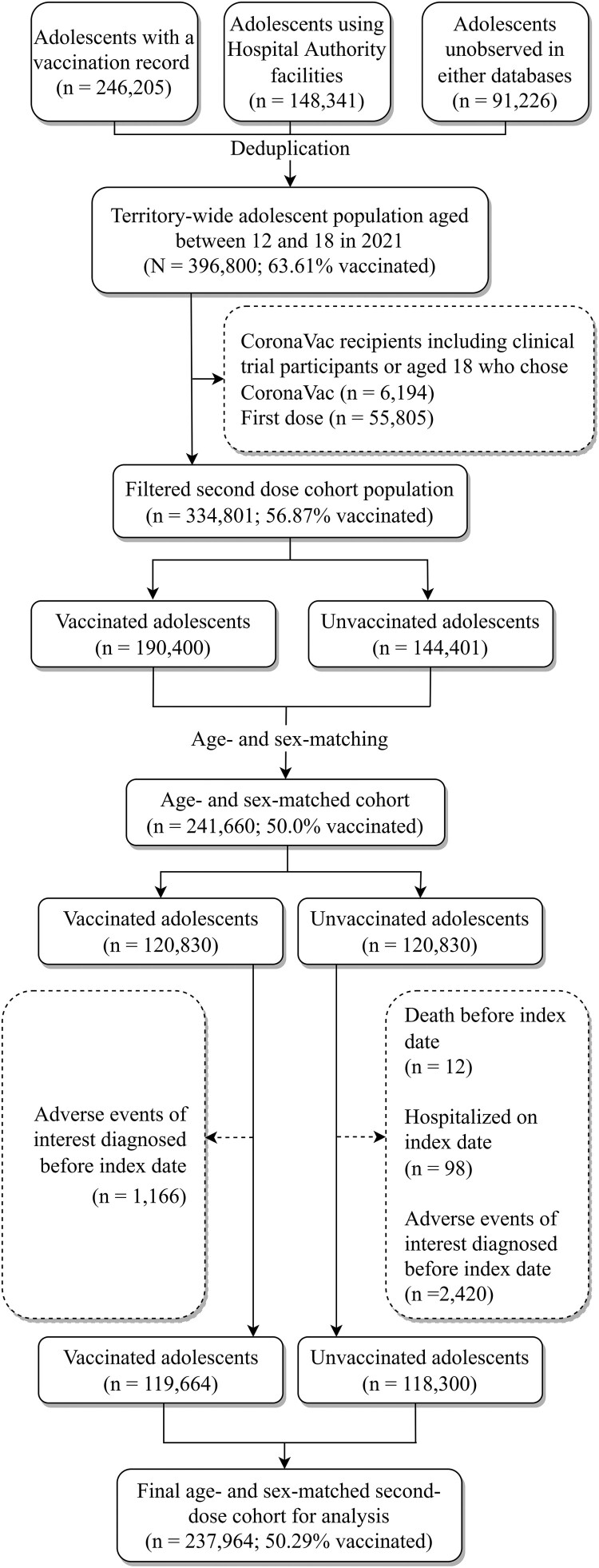
Flowchart of second-dose cohort selection.

For the first-dose cohort, after age- and sex-matching for mapping the index date from the unvaccinated to the vaccinated group, 278,964 individuals remained. Eventually, the cohort comprised 274,884 patients, with 50.25% of the adolescents vaccinated at least one dose after subsequent removal of ineligible individuals. Following the same selection procedures, the final second-dose cohort consisted of 237,964 individuals with 50.29% vaccinated with two doses. Figure 1 displays the flowchart of the cohort selection for the first-dose cohort and Figure 2 shows that for the second-dose cohort.

### Cohort characteristics: age and sex

The cohort characteristics for both cohorts are summarized in Table 1. For the first-dose cohort, the male proportion of the vaccinated and unvaccinated groups was 50.36%% and 50.27%, respectively. For the second-dose cohort, it was 51.12% and 51.02%. The mean age was highly similar between the vaccinated and unvaccinated in both cohorts.

**Table 1.** Sex and age distribution between the vaccinated and unvaccinated groups.

	Vaccinated	Unvaccinated
First-dose cohort		
Sex		
Male	69,572 (50.36%)	68,747 (50.27%)
Female	68,569 (49.64%)	67,996 (49.73%)
Age		
*M* (*SD*)	14.170 (1.821)	14.157 (1.816)
12	28,008	28,907
13	33,040	32,884
14	23,324	23,134
15	20,044	19,863
16	11,951	11,674
17	12,443	12,154
18	8331	8127
Second-dose cohort		
Sex		
Male	61,168 (51.12%)	60,352 (51.02%)
Female	58,496 (48.88%)	57,948 (48.98%)
Age		
*M* (*SD*)	14.440 (1.803)	14.426 (1.799)
12	18,300	18,235
13	25,461	25,355
14	23,091	22,885
15	20,063	19,871
16	11,954	11,677
17	12,455	12,153
18	8340	8124

### Adverse events of special interest

**Figure 3.**
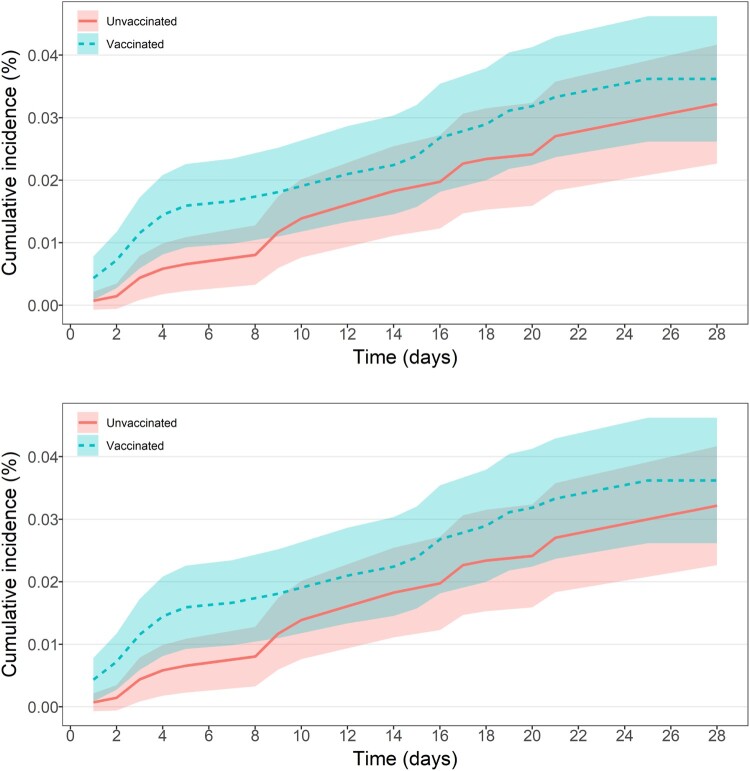
(a) Cumulative incidence with 95% confidence interval (shaded area) of any AESI of first-dose vaccinated and unvaccinated groups within 28-day observation period. (b) Cumulative incidence with 95% confidence interval (shaded area) of any AESI of second-dose vaccinated and unvaccinated groups within 28-day observation period.

Over the observation period for the first-dose cohort, 94 adolescents experienced at least one AESI, constituting an overall incidence of 0.3 per 1000 persons [95% confidence interval (CI) 0.3–0.4]. For the second-dose cohort, the incidence was 0.5 (95% CI 0.5–0.6). Figure 3 shows that the estimated cumulative incidence (of any AESI) over the observation period, with the vaccinated group largely overlapping with the unvaccinated in the first-dose cohort. Nevertheless, an elevated cumulative incidence of AESI was observed among the vaccinated group compared with the unvaccinated group for the second-dose cohort.

For the first-dose cohort, the most common AESI was sleeping disturbance/disorder (*n* = 18 for the vaccinated group; *n* = 16 for the unvaccinated group), followed by anaphylaxis (*n* = 8 for vaccinated group; *n* = 7 unvaccinated group), myocarditis (*n* = 8 for vaccinated group; *n* = 1 for unvaccinated group), and arrhythmia (*n* = 7 for vaccinated group; *n* = 2 for unvaccinated group). Likewise, for the second-dose cohort, the most common AESI was sleeping disturbance/disorder (*n* = 23 for the vaccinated group; *n* = 11 for the unvaccinated group), followed by myocarditis (*n* = 30 for vaccinated group; *n* = 1 for unvaccinated group), and arrhythmia (*n* = 12 for vaccinated group; *n = *6 for unvaccinated group).

### Poisson regression model

As shown in Table 2, Poisson regression analyses suggested that, for the first-dose cohort, there were no marked differences in the risk of any AESI upon receiving the vaccine during the observation period of 28 days except for myocarditis (IRR = 9.15, 95% CI 1.14–73.16, *P *= 0.037). Similarly, the sub-category analyses indicated that the vaccinated group had a five-fold higher incidence rate of 5.34 (95% CI 1.53–18.56) in the cardiovascular system AESIs compared with the unvaccinated group (*p *= 0.009). All other sub-category analyses suggested no significant differences in the association of vaccination status with any other types of AESIs (*P* > 0.05). The incidence of myocarditis was estimated at 2.91 (95% CI 1.26–5.73) per 100,000 vaccinated persons compared with 0.36 (95% CI 0.01–2.03) among the unvaccinated.

**Table 2.** Incidence rate ratios with 95% confidence intervals of adverse events of special interest (AESI) from age- and sex-adjusted Poisson regressions censoring on receiving second dose and 28-day of first dose inoculation.

Adverse events of special interest (AESI)	Vaccinated	Unvaccinated	IRR	2.50%	97.50%	*p*-value
***Any AESI***	*50*	*44*	*1.2999*	*0.8669*	*1.9492*	*0.2044*
**Autoimmune diseases**	2	1	2.2876	0.2074	25.2279	0.4993
Acute disseminated encephalomyelitis (ADEM)	0	0	–	–	–	–
Acute aseptic arthritis	1	0	–	–	–	–
Guillain-Barre syndrome	0	0	–	–	–	–
Idiopathic thrombocytopenia	0	0	–	–	–	–
Subacute thyroiditis	0	0	–	–	–	–
Type 1 diabetes	1	1	1.1434	0.0715	18.2803	0.9245
**Cardiovascular system**	14	3	**5.3363**	**1.5342**	**18.5610**	**0.0085**
Arrhythmia	7	2	3.9993	0.8308	19.2512	0.0838
Coronary artery disease	0	0	–	–	–	–
Heart failure	0	0	–	–	–	–
Microangiopathy	0	0	–	–	–	–
Myocarditis	8	1	**9.1507**	**1.1445**	**73.1606**	**0.0369**
**Circulatory system**	3	1	3.4296	0.3568	32.9708	0.2858
Haemorrhagic disease	2	1	2.2880	0.2075	25.2322	0.4992
Single organ cutaneous vasculitis	1	1	1.1432	0.0715	18.2766	0.9246
Thromboembolism	1	0	–	–	–	–
**Hepato-renal system**	1	6	0.1906	0.0229	1.5829	0.1248
Acute kidney injury	1	3	0.3811	0.0396	3.6635	0.4034
Acute liver injury	0	3	–	–	–	–
Acute pancreatitis	0	0	–	–	–	–
**Nerves and central nervous system**	4	10	0.4576	0.1435	1.4590	0.1864
Bell's Palsy	1	3	0.3813	0.0397	3.6654	0.4037
Generalized convulsion	3	6	0.5721	0.1431	2.2868	0.4295
Meningoencephalitis	0	1	–	–	–	–
Transverse myelitis	0	0	–	–	–	–
**Respiratory system**	0	2	–	–	–	–
Acute respiratory distress syndrome	0	2	–	–	–	–
**Skin and mucous membrane, bone, and joints system**	0	0	–	–	–	–
Chilblain like lesions	0	0	–	–	–	–
Erythema multiforme	0	0	–	–	–	–
**Other systems**	26	23	1.2932	0.7379	2.2663	0.3691
Anaphylaxis	8	7	1.3069	0.4739	3.6040	0.6050
Anosmia, ageusia	0	0	–	–	–	–
Multisystem inflammatory syndrome	0	0	–	–	–	–
Sleeping disturbance/disorder	18	16	1.2871	0.6564	2.5239	0.4626
Rhabdomyolysis	0	0	–	–	–	–

For the second-dose cohort as shown in Table 3, a similar pattern was observed and the IRR for myocarditis was estimated at 29.61 (95% CI 4.04–217.07) and that for cardiovascular system AESIs at 5.92 (95% CI 2.66–13.17). In addition, the IRR for sleeping disturbance/disorder was estimated at 2.06 (95% CI 1.01–4.24). The incidence of myocarditis was estimated at 12.61 (95% CI 8.51–18.00) per 100,000 vaccinated persons compared with 0.42 (95% CI 0.01–2.34) among the unvaccinated.

**Table 3.** Incidence rate ratios with 95% confidence intervals of adverse events of special interest (AESI) from age- and sex-adjusted Poisson regressions comparing two-dose vaccination with non-vaccination censoring on 28-day of second dose inoculation.

Adverse events of special interest (AESI)	Vaccinated	Unvaccinated	IRR	2.50%	97.50%	*p*-value
***Any AESI***	*84*	*46*	***1.8012***	***1.2572***	***2.5804***	***0.0013***
**Autoimmune diseases**	3	5	0.5923	0.1416	2.4783	0.4733
Acute disseminated encephalomyelitis (ADEM)	0	0	–	–	–	–
Acute aseptic arthritis	1	0	–	–	–	–
Guillain-Barre syndrome	0	0	–	–	–	–
Idiopathic thrombocytopenia	0	2	–	–	–	–
Subacute thyroiditis	1	0	–	–	–	–
Type 1 diabetes	1	3	0.3277	0.0341	3.1500	0.3339
**Cardiovascular system**	42	7	**5.9163**	**2.6584**	**13.1664**	**0.0000**
Arrhythmia	12	6	1.9682	0.7387	5.2442	0.1757
Coronary artery disease	1	0	–	–	–	–
Heart failure	0	0	–	–	–	–
Microangiopathy	0	0	–	–	–	–
Myocarditis	30	1	**29.6128**	**4.0398**	**217.0686**	**0.0009**
**Circulatory system**	2	4	0.4944	0.0906	2.6995	0.4161
Haemorrhagic disease	0	3	–	–	–	–
Single organ cutaneous vasculitis	1	0	–	–	–	–
Thromboembolism	1	1	0.9833	0.0615	15.7215	0.9905
**Hepato-renal system**	0	4	–	–	–	–
Acute kidney injury	0	1	–	–	–	–
Acute liver injury	0	3	–	–	–	–
Acute pancreatitis	0	0	–	–	–	–
**Nerves and central nervous system**	10	11	0.8936	0.3795	2.1040	0.7968
Bell's Palsy	4	2	1.9598	0.3590	10.6981	0.4371
Generalized convulsion	6	8	0.7379	0.256	2.1266	0.5735
Meningoencephalitis	0	1	–	–	–	–
Transverse myelitis	0	0	–	–	–	–
**Respiratory system**	1	0	–	–	–	–
Acute respiratory distress syndrome	1	0	–	–	–	–
**Skin and mucous membrane, bone, and joints system**	1	0	–	–	–	–
Chilblain like lesions	1	0	–	–	–	–
Erythema multiforme	0	0	–	–	–	–
**Other systems**	33	18	**1.8097**	**1.0190**	**3.2138**	**0.0429**
Anaphylaxis	8	7	1.1282	0.4092	3.1105	0.8157
Anosmia, ageusia	0	0	–	–	–	–
Multisystem inflammatory syndrome	2	0	–	–	–	–
Sleeping disturbance/disorder	23	11	**2.0647**	**1.0065**	**4.2355**	**0.0480**
Rhabdomyolysis	1	0	–	–	–	–

### Sensitivity analysis

With myocarditis excluded from the composite AESI outcome for the second-dose cohort, no significant association between vaccination and AESI was observed (IRR = 1.29, 95% CI 0.87–1.90, *P *= 0.195).

## Discussion

This study is one of the first cohort studies describing and comparing AESIs after receiving BNT162b2 among adolescents. Overall, the BNT162b2 is found to have an acceptable safety profile for adolescents in Hong Kong, as evidenced by a very low incidence of AESIs following vaccination without a marked elevation of AESI risk compared with the unvaccinated, except there is an increased risk of myocarditis, especially following the second dose. A marginally significant heightened risk of sleeping disturbance/disorder was also observed following the second dose.

Compared with previous research in Western populations, the observed absolute risk of myocarditis following BNT162b2 use, especially the second dose, was apparently higher i.e. above 10 per 100,000 vaccinated persons. For instance, in a recent Israeli study, among those aged 16–29, the cumulative incidence over 42 days was estimated at 5.49 per 100,000 vaccinated individuals. In a British study, only one additional event per one million persons was estimated to be induced by the use of BNT162b2 [10]. This difference may have arisen from a potential underestimation of the risk in the Western populations, a younger cohort adopted in this current study, or unknown genetic differences between the Chinese and Western populations. These speculations warrant further research to substantiate or refute.

A recent study conducted in Hong Kong examining the risk of myocarditis and pericarditis following BNT162b2 vaccination showed that there was an overall increased risk of myocarditis among adolescents (overall incidence rate 18.52 (95% CI 11.67–29.09) per 100,000 persons vaccinated). Amongst the 33 cases described, all had mild diseases and recovered spontaneously. The risk was particularly prominent among males after the second dose [incidence rate 37.32 (95% CI 26.98–51.25) per 100,000]. All cases were mild, and did not require inotropic, ventilatory and circulatory support [18]. This was consistent with the observation in this study, as well as in other jurisdictions, including the United States [28–31] and Israel [7,11]. A recent murine model study has demonstrated the association between mRNA vaccine and myopericarditis, probably because of higher systemic levels of mRNA lipid nanoparticles due to inadvertent intravenous injection or rapid return from the lymphatic circulation [32]. This finding, together with our current observations, provides evidence to support the recommendation for adolescents between 12 and 17 years in Hong Kong (since 16 September 2021) to receive one dose of BNT162b2 only [27]. These adolescents who suffered from vaccine-related myocarditis shall warrant long-term follow-up for any potential cardiovascular sequelae.

Despite the increased risk of myocarditis and the marginally increased risk of sleeping disturbance/disorder the other AESIs recorded among the vaccinated group were similar to the unvaccinated group. Such observation was consistent with local studies conducted in the adult population [16,17]. Narcolepsy, one of the conditions covered by our definition of sleeping disturbance/disorder, has not been reported in other studies on COVID-19 vaccine-related AESI so far, but reported previously to be possibly associated with influenza vaccination [33]. In our study, no patients coded as having sleeping disturbance/disorder had this particular condition (ICD-9 347.xx). Contrary to observation in the West, our study did not reveal an increased risk of thromboembolism following BNT162b2 vaccination [34], which is likely due to a younger population and possibly the inherently lower risk of thromboembolism among Asians to Caucasians [35]. However, this absence of risk elevation might be due to insufficient statistical power as well. Continuous safety monitoring is still needed.

This study has several limitations. First, adolescents who experienced AESIs may have sought medical attention in the private sector. Nevertheless, data collected in this study is considered representative as the HA constitutes approximately 80% of the inpatient services in Hong Kong [36]. Second, in common with all observational studies, residual confounders cannot be completely excluded in this retrospective database analysis. Third, similar to other large-scale pharmacovigilance studies using electronic medical record databases, we only relied on the diagnostic codes and other records for the operationalization of the diseases despite the demonstrated accuracy of those codes [37]. Fourth, the number of adolescents not using HA services and without a vaccination record was calculated using the C&SD population estimates by age and sex. The validity of the study results is therefore subject to the accuracy of those numbers. Last, as the population of Hong Kong is predominantly Chinese, replication of the analyses in other world populations is warranted to test for the generalizability of the results.

## Conclusion

Adolescents receiving BNT162b2 vaccine had an increased risk of myocarditis and sleeping disturbance/disorder, but not other AESIs. The overall risk of COVID-19 infection in different jurisdictions should be weighed against these specific risks in adolescents.

## Supplementary Material

Supplemental Material

Supplemental Material

## Data Availability

Data will not be available for third parties as permission has not been obtained from the data custodians.
