# Supplementary material for: Adverse events of special interest following the use of BNT162b2 in adolescents: a population-based retrospective cohort study
Source: Emerg Microbes Infect. 2022 Mar 21;11(1):885–93. doi: 10.1080/22221751.2022.2050952 (PMC8942549; doi:10.1080/22221751.2022.2050952)
Supplement: Supplemental Material [file TEMI_A_2050952_SM0050.docx]

**Captions of Figures, Tables and Supplementary Tables**

Figure 1. *Flowchart of first-dose cohort selection*

Figure 2. *Flowchart of second-dose cohort selection*

Figure 3a. *Cumulative incidence with 95% confidence interval (shaded area) of any AESI of first-dose vaccinated and unvaccinated groups within 28-day observation period*

Figure 3b. *Cumulative incidence with 95% confidence interval (shaded area) of any AESI of second-dose vaccinated and unvaccinated groups within 28-day observation period*

Table 1. *Sex and age distribution between the vaccinated and unvaccinated groups*

Table 2. *Incidence rate ratios with 95% confidence intervals of adverse events of special interest (AESI) from age- and sex-adjusted Poisson regressions censoring on receiving second dose and 28-day of first dose inoculation*

Table 3. *Incidence rate ratios with 95% confidence intervals of adverse events of special interest (AESI) from age- and sex-adjusted Poisson regressions comparing two-dose vaccination with non-vaccination censoring on 28-day of second dose inoculation*

eTable 1. *List of adverse events of interest and corresponding diagnosis codes*
