# Supplementary material for: Adverse events of special interest following the use of BNT162b2 in adolescents: a population-based retrospective cohort study
Source: Emerg Microbes Infect. 2022 Mar 21;11(1):885–93. doi: 10.1080/22221751.2022.2050952 (PMC8942549; doi:10.1080/22221751.2022.2050952)
Supplement: Supplemental Material [file TEMI_A_2050952_SM0044.docx]

eTable 1. *List of adverse events of interest and corresponding diagnosis codes.*

| Adverse events of interest | ICD-9 codes | ICPC codes |
| --- | --- | --- |
| Auto-immune diseases |  |  |
| Guillain-Barré Syndrome | 357.0, 357.8x, 357,9 | N94, N94005 |
| Acute disseminated encephalomyelitis (ADEM) | 323.6x, 323.8x |  |
| Sleeping disturbance/disorder | 347.xx, 89.17, 89.18, 307.4, 780.5 |  |
| Acute aseptic arthritis | 274.0x, 696.0, 716.5x, 716.6x, 716.9x, 712.xx, 711.5x |  |
| Type 1 Diabetes | 250.01, 250.03, 250.11, 250.13, 250.21, 250.23, 250.31, 250.33, 250.41, 250.43, 250.51, 250.53, 250.61, 250.63, 250.71, 250.73, 250.81, 250.83, 250.91, 250.93 | T89 |
| (Idiopathic) Thrombocytopenia | 287.3x, 287.4x, 287.5, 279.12, 283.11, 284.1x, 446.6, 776.1 |  |
| Subacute thyroiditis | 245.1 |  |
| Cardiovascular system diseases |  |  |
| Microangiopathy | 446.6 |  |
| Heart failure | 428.xx, 398.91, 402.01, 402.11, 402.91, 404.01, 404.03, 404.11, 404.13, 404.91, 404.93 | K77 |
| Stress cardiomyopathy | 429.83 |  |
| Coronary artery disease | 410.xx-414.xx, V45.81, 36.0x, 36.1x, 36.2x, 36.3x | K74, K75, K76 |
| Arrhythmia | 427.xx, 426.7, 624.x, 794.3x, 785.0 | K79, K80 |
| Myocarditis | 422.xx, 429.0, 420.9, 423.9 | K84, K84010 |
| Circulatory system diseases |  |  |
| Thromboembolism | 415.1x, 453.xx, 443.xx, 444.xx, 445.xx, 433.xx, 434.xx, 435.xx, 436, 437.0, 437.1, 437.6, 437.8, 437.9, 451.x, 452.x, 325, 286.6x, 459.9, 434.01, 557.0, 557.9, 453.40, 453.41, 453.42, 410.x | K74, K75, K76, K89, K90, K91, K93, K94 |
| Hemorrhagic disease | 286.5x, 286.7, 287x, 430, 431, 432.0, 432.9 | K90, K91 |
| Single Organ Cutaneous Vasculitis | 709.1, 446.2x, 287.0 | B83, K99, B83019, K99016 |
| Hepato-renal system diseases |  |  |
| Acute liver injury | 570.xx, 573.3 | D80, D97 |
| Acute kidney injury | 584.xx, 586.xx | U99, U99005 |
| Acute pancreatitis | 577.0 |  |
| Nerves and central nervous system diseases |  |  |
| Generalized convulsion | 345.xx, 780.3x, 779.0, 333.2, 649.4 | N07, N88 |
| Meningoencephalitis | 322.9x, 323.0x, 323.4x, 323.5x, 323.6x, 323.8x, 323.9x, 323.7, 330.8, 337.73, 046.3, 049.8, 036.1, 056.01, 136.2, 130, 054.3, 049.0, 094.1, 072.2, 013.0, 062.4, 049.9, 045.0, 062 | N71, N70 |
| Transverse myelitis | 323.0x, 323.4x, 323.5x, 323.6x, 323.8x, 341.2x |  |
| Bell’s Palsy | 351.0, 351.8, 351.9 | N91 |
| Respiratory system disease |  |  |
| Acute respiratory distress syndrome | 518.8x, 518.5x, 96.7x | R99, R99004 |
| Skin, bone, and joints system diseases |  |  |
| Erythema multiforme | 695.1x |  |
| Chilblain – like lesions | 991.5 | A88, A88001 |
| Other system diseases |  |  |
| Anosmia, ageusia | 781.1 | N16 |
| Anaphylaxis | 995.0, 995.1, 995.3, 995.4, 999.4x, 708.9, 519.11, 786.1, 458.9, 519.1x | A12, A99, A84, A85 |
| Multisystem inflammatory syndrome in children | 446.1 | B99, B99022 |
| Rhabdomyolysis | 728.88, 728.89, 791.3 |  |
